# Chicago Gynæcological Society

**Published:** 1885-07-20

**Authors:** 


### CHICAGO GYNÆCOLOGICAL SOCIETY.

Dr. Henry T. Byford read a paper entitled "Functions of the Membranes in Labor."

Dr. Byford discussed fairly, clearly, and forcibly the possibility, probability, and utility of the preservation of the membranes from rupture until they dilate, or aid in dilating the vulvo-vaginal orifice, as well as the cervix. The possibility and probability are not only matters of reason, but, under the given appropriate management, have stood the test of experience in the author's practice.

The utility lies in the substitution of a soft bag for the hard, rough, unyielding head as a dilator; in the retention of the amniotic fluid in the parturient canal as a cushion, protector, and lubricator; but chiefly in the *manner* of dilating the vulva and perineum, viz, by making the axis of the vulva correspond with the axis of the presenting part *before* its exit. The perineum is stretched along its lateral diameters only, while the fourchette descends to within half an inch of the anus. This brings the transverse muscular fibres of the perineum nearer together, instead of further apart, and renders their rupture a matter of proportionate difficulty. All artificial interference in dilating the perineum should be based upon this mechanism.

Dr. W. W. Jaggard, while not prepared to accept the thesis of the paper as a universal proposition, thought the attention to the functions of the membranes advised by Dr. Byford would go far to prevent cervical, vaginal, and perineal lacerations in many cases.

Dr. D. T. Nelson said the membranes, when adherent to the cervix, as pointed out by Dr. DeLaskie Miller, delayed labor. Under these conditions puncture of the membranes is indicated. In practice he tried to find out whether or no the membranes were adherent to the cervix. If adherent, he punctured the membrane; if free, he always waited for spontaneous rupture.

Dr. Philip Adolphus agreed with the author of the paper in his conclusions, except in case of hydramnion. Under such pathological conditions it was frequently necessary to rupture the membranes, in order to secure uterine contractions.

Dr. J. Suydam Knox was in the habit of aiding dilatation of the vaginal os with the finger. He was under the impression that the membranes were seldom operative in the dilatation of the vulvo-vaginal orifice.

Dr. Edmund J. Doering had followed Dr. Byford's suggestion during the past year, and believed the method had prevented perineal lacerations in many cases. Premature detachment of the placenta was a possible danger from delayed rupture of the bag of membranes.

Dr. Edward W. Sawyer believed that the membranes should not be punctured until the os uteri was dilated to the extent which the pelvis, in the concrete case, would permit. The function of the bag of waters in labor was the dilatation of the os uteri. He ruptured the amnion in one-half his cases. There was as great variation in the development of the uterine muscular tissue as in the dovelopment of the biceps. Retardation of rupture of the bag of membranes might lead to uterine inertia, or to premature detachment of the placenta. To follow Nature's suggestions was a beautiful theory ; but Nature caused the membranes to be ruptured, in a majority of cases, before dilatation of the vaginal os. He was in the habit of aiding dilatation of the cervix by the introduction of one or two fingers within the canal of the cervix ; he also tried to peel off the membranes around the internal os, and was conscious that he had materially influenced, in a favorable direction, the process of parturition. The periphery of the os uteri was less than the periphery of the vaginal orifice. It was inconceivable, under such physical conditions, that the bag of membranes, protruding through a smaller, could dilate a larger orifice.

Dr. Charles W. Earle agreed with Dr. Sawyer, that natural processes should be imitated in the puncture of the bag of membranes after complete dilatation of the os uteri.

Dr. H. P. Merriman thought Dr. Byford's paper contained excellent advice for the young practitioner. The thesis could not be accepted as a universal proposition. Dr. Sawyer and Dr. Earle had pointed out the conditions in which the application of the method was contraindicated.

Dr. Henry T. Byford, in conclusion, said that he had distinctly pointed out in his paper that the retardation of rupture of the membranes was advisable only in perfectly physiological labors. The members of the Society called attention to pathological conditions to which the method did not apply.

Dr. Sawyer had read a paper on "Occipito-posterior Positions," before the American Gynæcological Society, during October. A plausible explanation of the fact that anterior rotation did not occur in Dr. Sawyer's thirty-nine cases might be found in the premature rupture of the membranes.

Dr. Byford claimed that the natural processes were not imitated, but interfered with, by rupturing the membranes at the complete dilatation of the os uteri.—*Jour. Am. Med. Association.*

